# Lychee Seed as a Potential Hypoglycemic Agent, and Exploration of its Underlying Mechanisms

**DOI:** 10.3389/fphar.2021.737803

**Published:** 2021-10-08

**Authors:** Yuehong Zhang, De Jin, Xuedong An, Liyun Duan, Yingying Duan, Fengmei Lian

**Affiliations:** ^1^ Department of Endocrinology, Guang’anmen Hospital, China Academy of Chinese Medical Sciences, Beijing, China; ^2^ Clinical department of Traditional Chinese Medicine, Beijing University of Chinese Medicine, Beijing, China

**Keywords:** lychee seed, diet supplements, diabetes, pharmacological mechanisms, effect

## Abstract

Food is people’s primal want. A reasonable diet and healthy food not only provide nutrients for human growth but also contribute to disease prevention and treatment, while following an unhealthy diet can lead to an increased risk of many diseases, especially metabolic disorders, such as diabetes. Nature is enriched with different food sources, and it seems that purely natural products are more in line with the current concept of health, which enhance the formation of the notion that “Food/Diet Supplements from Natural Sources as a Medicine.” As a delicious fruit, the medicinal values such as anticancer, antibacterial, antioxidation, and antiglycating properties of lychee have been found. Lychee (*Litchi* in Chinese) is a subtropical fruit plant belonging to the family Sapindaceae. It has been widely cultivated in warm climates worldwide, particularly in China, for thousands of years. In recent years, various phytochemical components such as quercetin, procyanidin A2, and (2R)-naringenin-7-O-(3-O-αL-rhamnopyranosyl-β-D-glucopyranoside) have been identified in a lychee seed, which may lend a lychee seed as a relatively safe and inexpensive adjuvant treatment for diabetes and diabetic complications. In fact, accumulating evidence has shown that lychee seed, lychee seed extracts, and related compounds have promising antihyperglycemic activities, including improving insulin resistance, anti-inflammatory effect, lipid regulation, neuroprotection, antineurotoxic effect, and renoprotection effect. In this review, we summarized publications on antiglycemic effects and mechanisms of lychee seed, lychee seed extracts, and related compounds, which included their efficacies as a cure for diabetes and diabetic complications in cells, animals, and humans, attempting to obtain a robust evidence basis for the clinical application and value of lychee seed.

## Introduction

Diabetes is a severe, long-term (or chronic) disease in the world, defined as a blood glucose profile higher than normal, due to a disturbed insulin secretion or a disturbed insulin effect or usually both (Petersmann et al., 2019). Based on the most recent data issued by the International Diabetes Federation (IDF) (Saeedi et al., 2019), the number of adults aged 20–79 years globally with diabetes has reached nearly 463.0 million in 2019. It is estimated that the number will rise to 578.4 million by 2030, and 700.2 million by 2045, which means that the global diabetes epidemic markedly increases at an incredible speed among populations. Obviously, it has become a significant global public health problem. Individuals with diabetes are more prone to develop complications such as retinopathy, nephropathy, coronary artery disease, peripheral arterial disease, and stroke, contributing to higher mortality rates (Ford, 2011; Tandon et al., 2012). Thus, the prevention and treatment of diabetes hold considerable importance. Currently, the main treatments for diabetes include insulin injection, oral diabetes medications, and pancreatic islet transplantation (Ryan et al., 2005; Doyle-Delgado et al., 2020). However, the available treatments only delay the progress of the disease rather than curing it, leading to the lengthy and costly therapy, and comprise side effects, which impart a heavy economic and psychological burden on patients.

Food is the first necessity of people. Poor diet is associated with a higher risk of many diseases (GBD 2017 Diet Collaborators, 2019), especially diabetes (Srour et al., 2020), while some healthy food is reported to improve glycemic control (Reynolds et al., 2020). Studies supported a positive association between dietary intake of momordica charantia and blood sugar reduction (Kibiti and Afolayan, 2015). Buckwheat also had effects on reducing serum glucose concentrations in diabetic rats (Kawa et al., 2003). Consequently, the notion that “Food/Diet Supplements from Natural Sources as a Medicine” has become popular and appealing among diabetic patients. In China, herbs in nature with homology of medicine and food have been widely studied. Lychee, a fruit tree belonging to family Sapindaceae, originating from China, is widely cultivated in warm climates in many regions around the world and is botanically related to Litchi chinensis Sonn (Jiang, 2003; Yang et al., 2006; Huang et al., 2014). Due to the high nutrients and savory flavor as well as the attractive red, lychee is widely favored by humans. Lychee seed, the dried mature seed of lychee, an ancient traditional drug–food homologous herbal medicine, was used to smooth Qi, dispel cold, alleviate polydipsia, and relieve pain in China (Kilari and Putta, 2016). Accumulating research studies recently focused on the antidiabetic activity of lychee seed, although the underlying mechanisms of action have not been studied thoroughly. A summary of antidiabetic studies of lychee seed will be helpful for offering a reference basis for deeper investigations and clinical use of this natural herb medicine (Table 1). We examined the electronic resources with the PubMed, EMBASE, Web of Science, and China National Knowledge Infrastructure based on the information limited to English and Chinese literatures up till Jun 2021.

**TABLE 1:** The antihyperglycemic activity and the mechanisms of lychee seed in clinical trials, *in vitro*, and *in vivo* studies.

Activities	Pharmacological effects	Model	Material	Dose/concentration (route of administration)	Duration	Species/exposure	Reference
Anti-diabetes	Decreased FPG	In vivo	Extract tablets of lychee seed	30 g/d (p.o., *n* = 45)	12 weeks	Patients with T2D	Zhang and Teng (1985)
	Lowered blood glucose and ameliorated symptoms	In vivo	Extract tablets of lychee seed water	3.6–5.4 g/d (p.o., *n* = 30)	12 weeks	Patients with T2D	Shen (1991)
	Lowered blood glucose	In vivo	Lychee seed ethanol extract	160 mg/kg/d (i.g., *n* = 10)	2 weeks	STZ-diabetic Wistar rats (220 ± 20 g)	Ren (2011)
	Lowered blood glucose	In vivo	Lychee seed extract fluid	0.1 ml/d (i.g., *n* = 20)	15 days	Alloxan induced diabetic mice (30–40 g)	Guo et al. (2013)
	Lowered blood glucose	In vivo	Lychee seed concentrated decoction	20 g/kg/d (i.g., *n* = 10)	10 days	Alloxan-diabetic Kunming mice (18–22 g)	Guo et al. (1999)
	Lowered blood glucose	In vivo	Total saponins of lychee seed	500 mg/kg/d (i.g.,*n* = 8)	2 weeks	Alloxan-diabetic Kunming male mice (18–20 g)	Yuan et al. (2006)
	Lowered blood glucose	In vivo	The alcohol extracted fract from lychee seed	300 mg/kg/d (i.g., *n* = 8)	2 weeks	High-fat/high-sucrose diet- diabetic SD rats (220 ± 20 g)	Jiang (2011)
	Lowered blood glucose	In vivo	The alcohol extracted fract from lychee seed	300 mg/kg/d (i.g., *n* = 8)	2 weeks	STZ-diabetic SD rats (220 ± 20 g)	Jiang et al. (2011)
	Lowered blood glucose	In vivo	Dry extract of lychee seed	2.6 mg/kg/d (i.g., *n* = 11)	30 days	Alloxan-diabetic Wistar rats (200 g)	Shen et al. (1986)
	Lowered blood glucose	In vivo	Lychee seed extract fluid	0.4 ml/d (containing 0.04 g crude drug,i.g., *n* = 14)	7 days	Alloxan-diabetic Kunming male mice (22–31 g)	Liang et al. (2009)
	Lowered blood glucose	In vivo	Lychee seed extract fluid	0.2 ml/d (i.g., *n* = 9)	7 days	Alloxan-diabetic Kunming mice (23–28 g)	Li et al. (2008)
	Lowered FBG and 2 h BG after OGTT; Improved IGT; Lowered FSG	In vivo	Saponin of lychee seed	0.2 g/kg/d (i.g., *n* = 12)	7 days	DX-induced insulin resistant SD rats (150–180 g)	Guo et al. (2003b)
	Displayed α-glucosidase inhibitory activity	In vitro	Total flavonoids of lychee seed	1 mg/ml	None	α -glucosidase inhibitory assay	Ren et al. (2017)
	Lowered FBG	In vivo	Lychee seed extract	0.3 ml (containing 0.015 g litchi seed extract, i.g.,n = 5)	12 weeks	db/db male mouse	Zhang et al. (2013)
	Lowered FBS	In vivo	Lychee seed extract fluid	0.4 ml/d (i.g., *n* = 12)	7 days	Alloxan-diabetic Kunming mice (22.5–25.5 g)	Chen et al. (2008)
	Displayed α-glucosidase inhibitory activity	In vitro	Lychee seed extract fluid	40 μL	None	α -glucosidase inhibitory assay	Zhou (2016)
	Displayed α-glucosidase inhibitory activity	In vitro	The crude extract, sugar-removed layer, pavetannin B2, procyanidin A2	IC_50_: 0.691 μg/ml, 3.686 μg/ml, 0.04 μM, and 0.08 μM	None	α -glucosidase inhibitory assay	Choi et al. (2017)
	Decreased FPG	In vivo	Water extract of lychee seed	0.1 ml/kg/d (i.g., *n* = 10)	10 days	Alloxan-diabetic Kunming mice	Kuang et al. (1997)
	Displayed α-glucosidase inhibitory activity	In vitro	Polysaccharide	IC_50_: 0.056 mg/ml	None	α -glucosidase inhibitory assay	Zhang et al. (2020)
	Displayed α-glucosidase inhibitory activity	In vitro	(2R)-Naringenin-7-O-(3-O-α-l-rhamnopyranosyl-β-d-glucopyranoside), (2S)-Pinocembrin-7-O-(6-O-α-Lrhamnopyranosyl-β-d-glucopyranoside)	1 mg/ml	None	α -glucosidase inhibitory assay	Ren et al. (2011)
	Reduced FBG and 1-h postprandial blood glucose	In vivo	Polysaccharides	400 mg/kg (i.g., *n* = 8)	30 days	Alloxan-diabetic ICR male mice (22–24 g)	Yuan (2010)
	Decreased FPG	In vivo	Lychee seed decoction	30 g/kg/d (i.g., *n* = 4)	2 weeks	Alloxan-diabetic SD male rats	Wu et al. (1991)
	Decreased FPG	In vivo	Total saponin extract from lychee seed	500 mg/kg/d (i.g., *n* = 10)	3 weeks	Alloxan-diabetic Kunming male mice (18–20 g)	Lou et al. (2007)
	Decreased FPG	In vivo	Lychee seed extract fluid	0.1 ml/d (i.g.,n = 8)	4 days	Alloxan-diabetic mice (20–24 g)	Chen et al. (2006)
	Displayed α-glucosidase inhibitory activity	In vitro	Semen lychee effective fractions	50 g/L	None	α -glucosidase inhibitory assay	Zhong et al. (2015)
	Inhibited the activities of both yeast and mammalian α-glucosidase	In vitro	Polysaccharide	IC_50_: 75.24 μM, 66.97 μM	None	Yeast (*Saccharomyces cerevisiae*) and mammalian (rat-intestinal acetone powder) α-glucosidase	Wu et al. (2020)
Improving insulin resistance	Reduced the levels of TNF-α, hyper-leptinemia, and FFA	In vivo	Lychee seed extract fluid	3.8 g/kg/d (i.g., *n* = 16)	31 days	STZ-diabetic SD rats (150–180 g)	Guo et al. (2004)
	Decreased the mRNA expression of RETN, PTP1B, and GRP78	In vitro	Semen lychee effective constituents	0.2 mg/ml	48 h	DX-induced insulin resistant 3T3–L1 cells	Liao et al. (2014)
	Inhibited the mRNA expression of GRP78 and CHOP	In vivo	Lychee semen effective constituents	0.47 g/kg/d (i.g., *n* = 8)	4 weeks	High-fat feeding combined with STZ-diabetic SD rats (180–200 g)	Li et al. (2015)
	Improved IR	In vivo	Lychee seed water extractant	3.8 g/kg/d (i.g., *n* = 16)	31 days	High caloric diet combined with STZ-diabetic SD rats (150–180 g)	Guo et al. (2003a)
	Reduced IRI; increased ISI	In vivo	Lychee seed extracts	30 mg/d (i.g., *n* = 6)	6 weeks	STZ/high-fat diet induced SD rats (100–130 g)	Man et al. (2016)
	Changed microRNAs expression	In vivo	Lychee seed extracts	0.015 g/d (i.g., *n* = 5)	12 weeks	db/db male mouse	Zhang et al. (2013)
Antioxidant effect	Increased the activity of SOD; decreased content of MDA	In vivo	Lychee seed extract fluid	3.8 g/kg/d (i.g., *n* = 16)	31 days	STZ-diabetic SD rats (150–180 g)	Guo et al. (2004)
	Improved activity of SOD; decreased the content of MDA	In vivo	Saponin of lychee seed	0.2 g/kg/d (i.g., *n* = 12)	7 days	DX-induced insulin resistant SD rats (150–180 g)	Guo et al. (2003b)
	Scavenge free radicals	In vitro	Total flavonoids of lychee seed	IC_50_: 0.00016 mg/ml	None	DPPH radical scavenging assay	Ren et al. (2017)
	Increased the activity of SOD; decreased content of MDA	In vivo	Lychee seed water and alcoholic extracts	62.50 g/kg/d(i.g., *n* = 12)	8 days	Alloxan-diabetic NIH mice(18–22 g)	Pan et al. (1999)
	Increased the level of GSH-PX; reduced the content of oxygen free radical	In vivo	Lychee seed extract fluid	0.1 ml/d (i.g., *n* = 8)	4 days	Alloxan-diabetic mice (20–24 g)	Chen et al. (2006)
	Accelerated the clearance of O_2_-	In vivo	Lychee seed extract fluid	0.5 ml/d (containing 0.012 g crude drug, i.g., *n* = 10)	7 days	Alloxan-diabetic Kunming male mice (25–30 g)	Li et al. (2006)
Anti-inflammatory effect	Downregulation expression of TGF-β1,MCP-1 and MIF	In vivo	Lychee semen effective constituents	6 g/kg/d (i.g., *n* = 10)	6 weeks	High-sugar/high-fat feeding SD male rats (200–220 g)	Qi (2017)
	Increased mRNA levels of NF-κB	In vivo	Lychee seed extracts	30 mg/d (i.g., *n* = 6)	6 weeks	(STZ)/high-fat diet induced SD rats (100–130 g)	Man et al. (2016)
	Inhibited the expression of MCP-1 and ICAM-1 protein in kidney tissue; reduced IL-1β and IL-6 in serum	In vivo	Saponin of lychee seed	2.5 mg/kg/d (i.g., *n* = 6)	8 weeks	STZ-diabetic Kunming male mice (18–22 g)	Qin (2017)
Lipid regulation	Lowered concentrations of TC and TG	In vivo	Lychee seed extract fluid	3.8 g/kg/d (i.g., *n* = 16)	31 days	STZ-diabetic SD rats (150–180 g)	Guo et al. (2004)
	Reduced serum TG level	In vivo	Lychee semen effective constituents	0.47 g/kg/d (i.g., *n* = 8)	4 weeks	High-fat feeding combined with STZ-diabetic SD rats (180–200 g)	Li et al. (2015)
	Reduced the content of TC, TG, and LDL-C	In vivo	Saponin of lychee seed	0.2 g/kg/d (i.g., *n* = 12)	7 days	DX-induced insulin resistant SD rats (150–180 g)	Guo et al. (2003b)
	Reduced the content of TC and TG; increased the content of HDL-C	In vivo	Lychee seed water and alcoholic extracts	62.50 g/kg/d (i.g., *n* = 12)	8 days	Alloxan-diabetic NIH mice (18–22 g)	Pan et al. (1999)
	Reduced the content of TC, TG; increased the content of HD L-C and ratio of HDL-C/TC	In vivo	Lychee seed water extractant	3.8 g/kg/d (i.g., *n* = 16)	31 days	High caloric diet combined with STZ-diabetic SD rats (150–180 g)	Guo et al. (2003a)
	Prevented the decrease of AMPK alpha 2 and p-AMPK levels; inhibited the synthesis of fatty acid, protein, and lipid metabolism	In vitro	Lychee semen active ingredients (LSE70 and LSE50)	2.5 μg/ml	24 h	HepG2 cell insulin resistance model	Man et al. (2019)
	Decreased TG, T-CHO, and LDH; increased the ratio of HDL-C to LDL-C	In vivo	Lychee seed extracts	30 mg/d (i.g., *n* = 6)	6 weeks	STZ/high-fat diet induced SD rats (100–130 g)	Man et al. (2016)
	Increased the content of HDL-C	In vivo	Lychee seed extract fluid	0.1 ml/d (i.g., *n* = 8)	4 days	Alloxan-diabetic mice (20–24 g)	Chen et al. (2006)
Kidney protection effect	Restrained the expression of HMC cell protein	In vitro	Saponin of lychee seed	20 mg/ml	48 h	HMC	Zhang (2016)
	Inhibited the proliferation of HBZY-1; reduced the protein level of TGF-β1, FN, and Col Ⅳ	In vitro	Total flavonoids of lychee	40 μg/ml	48 h	HBZY-1 induced by high glucose combined with TNF-α	Liu (2016)
	Inhibited the expression of MCP-1 and ICAM-1 protein; reduced the content of IL-1β and IL-6	In vivo	Saponin of lychee seed	2.5 mg/kg/d (i.g., *n* = 6)	8 weeks	STZ-diabetic Kunming male mice (18–22 g)	Qin (2017)
	Decreased the protein expression of FN and Col IV	In vitro	Total flavonoids of lychee	40 μg/ml	48 h	HBZY-1 induced by high glucose combined with TNF-α	Liu et al. (2016)
Neuroprotection and cognitive function improvement	Decreased Aβ and Tau deposition	In vivo	Lychee seed extract	2.78 g/kg/d (i.g., *n* = 12)	4 weeks	STZ-diabetic SD rats (180–220 g)	Zeng et al. (2016)
	Improved the transmit function of cholinergic nerve system in the cerebrum of mice	In vivo	Lychee seed extract fluid	0.5 ml/d (containing 0.012 g crude drug, i.g., *n* = 10)	7 days	Alloxan-diabetic Kunming male mice (25–30 g)	Li et al. (2006)
	Inhibited Tau hyperphosphorylation through improving IR *via* the IRS-1/PI3K/Akt/GSK-3β pathway	In vitro	Catechin, procyanidin A1, and procyanidin A2	10 μM	24 h	DX-induced HepG2 and HT22 cells	Xiong et al. (2020)
	Decreased Aβ, AGEs, and Tau protein	In vivo	Lychee seed extract	0.7 g/kg/d (i.g., *n* = 10)	28 days	High-fat/high-sugar/high protein feeding combined with STZ-diabetic rats	Tang et al. (2018)

Abbreviation: fasting plasma glucose (FPG); type 2 diabetes (T2D); streptozotocin (STZ); Sprague-Dawley (SD); fasting blood glucose (FBG); 2-h blood glucose (2 h BG); oral glucose tolerance test (OGTT); impaired glucose tolerance (IGT); serum contents of fasting glucose (FSG); Dexamethasone (Dx); Institute of Cancer Research (ICR); fasting blood sugar (FBS); tumor necrosis factor-α (TNF-α); free fatty acids (FFAs); insulin resistance (IR); insulin resistance index (IRI); insulin sensitivity index (ISI); National Institutes of Health (NIH); superoxide dismutase (SOD); malondialdehyde (MDA); 1,1-diphenyl-2-picrylhydrazyl (DPPH); glutathione peroxidase (GSH-Px); transforming growth factor-β1 (TGF-β1); monocyte chemotactic protein 1(MCP-1); macrophage migration-inhibitory factor (MIF); nuclear factor-κB (NF-κB); intercellular cell adhesion molecule-1(ICAM-1); interleukin-1β (IL-1β); interleukin-6 (IL-6); total cholesterol (TC); triglyceride (TG); low-density lipoprotein cholesterol (LDL-C); high-density lipoprotein cholesterol (HDL-C); Hepatocellular carcinoma cell (HepG2 cell); total cholesterol (T-CHO); lactate dehydrogenase (LDH); human glomerular mesangial cells (HMC); rat glomerular mesangial cells (HBZY-1); fibronectin(FN); collagen IV (Col IV); amyloid beta (Aβ); advanced glycation end products (AGEs).

## Botanical Descriptions of Lychee

**FIGURE 1:**
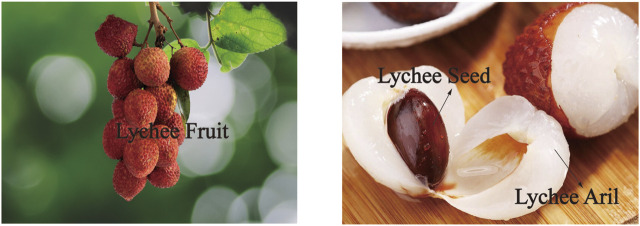
Lychee fruit, lychee aril, and lychee seed (color version of these figures is available at https://699pic.com/tupian-500450779.html?bindPhone=1, https://item.m.jd.com/product/53077629044.html?).

Lychee (*Litchi chinensis* Sonn.) is a subtropical medium evergreen dome-shaped tree with a glossy grayish stem belonging to the family Sapindaceae. It generally grows to less than 10 m in height, rarely up to 15 m or more. The pinnate leaves which consist of 4–8 pairs of elliptic or lanceolate, long acuminate, glabrous leaflets are leathery, 5–7 cm long, and 2–4 cm broad. The yellowish-white flower is small in size with a tetramerous calyx. Terminal inflorescence is about 5–30 cm long with multibranched panicles and slender pedicels. The fruit is ellipsoidal or nearly round in shape, estimated at 2.5 cm in diameter and clothed with a coarse thicker ring or pericarp with strawberry to red in color at maturity. Inside the pericarp is lychee aril that is milky-white and semitransparent with a sweet, juicy, and delicious taste. A seed with a smooth and glossy surface is brown or reddish brown in color and elliptic to ovate in shape covered by a fleshy aril. The size of the seed varies greatly between 1 and 2 cm in length, as shown in Figure 1.

## The History of Lychee Seed

Lychee seed, the dry mature seed of *Litchi chinensis* Sonn, is known to have a remarkable medicinal value in ancient China. The oldest available Chinese written source which described the application of lychee seed is Ben Cao Yan Yi traced back to the Song dynasty (AD 1116). In ancient Chinese medical practices, lychee seed was always used for hernia, orchitis, ulcers, and intestinal troubles. Diabetes-related symptoms were known as “Xiaoke” (emaciation and thirst) in the ancient Chinese medical literature (Tong et al., 2012). In Compendium of Materia Medica (Ben Cao Gang Mu) written by Li Shizhen (from 1518 to 1593 AD) during the Ming dynasty, the lychee seed was warm in nature, sweet in taste, and could act as a beneficial agent in thirst-quenching. Numerous Chinese patent medicines such as Jinlida granule (Tian et al., 2018) and Jiangtangtongmai tablets (Su et al., 2017) approved by the Chinese Food and Drug Administration, containing lychee seed, were clinically used for the treatment of diabetes.

## Potential Bioactive Compounds of Lychee Seed Responsible for Hypoglycemic Activities

Lychee seed is thought to improve glycemic control *via* various bioactive compounds with great pharmaceutical and biomedical potential. The flavanones, flavonols, proanthocyanidins, and dihydrochalcone fractions of lychee seed are the most investigated for their hypoglycemic activities. With the continuous optimization of the lychee seed extraction process, a number of monomers have been successfully identified and isolated. Here, several single isolated compounds including (2R)-naringenin-7-O-(3-O-αL-rhamnopyranosyl-β-d-glucopyranoside) (Ren et al., 2011), (2S)-Pinocembrin-7-O-(6-O-a-l-rhamnopyranosyl-β-d-glucopyranoside) (Ren et al., 2011), quercetin (Ren et al., 2013), procyanidin A1 (Xiong et al., 2020), procyanidin A2 (Choi et al., 2017), and phlorhizin (Ren et al., 2013) exhibiting potential beneficial effects on regulating glycemia are prominently described in Table 2.

**TABLE 2:** Antihyperglycemic compounds isolated from lychee seed.

Compound	Molecular formula	Reference
Flavanones		
2R)-Naringenin-7-O-(3-O-α-l-rhamnopyranosyl-β-d-glucopyranoside)	C_27_H_32_O_14_	Ren et al. (2011)
(2S)-Pinocembrin-7-O-(6-O-a-l-rhamnopyranosyl-β-d-glucopyranoside)	C_27_H_32_O_13_	Ren et al. (2011)
Flavonols		
Quercetin	C_15_H_10_O_7_	Ren et al. (2013)
Proanthocyanidins		
Procyanidin A1	C_31_H_28_O_12_	Xiong et al. (2020)
Procyanidin A2	C_30_H_24_O_12_	Choi et al. (2017), Xiong et al. (2020)
Dihydrochalcones		
Phlorhizin	C_21_H_24_O_10_	Ren et al. (2013)

## Pharmacology

### Improving Insulin Resistance

Insulin has a pivotal function in ensuring the homeostasis of energy metabolism through a coordination of the storage and utilization of fuel molecules in insulin-targeted organs (Castan-Laurell et al., 2012). Insulin resistance (IR) is a pathological condition defined by the inability of insulin to stimulate glucose disposal and is considered as a key player in the development of type 2 diabetes mellitus (Brown and Walker, 2016). Although the precise pathophysiology of IR in diabetes has not yet been delineated, inflammatory response, oxidative stress, insulin receptor mutations, endoplasmic reticulum stress, and mitochondrial dysfunction are currently regarded as the possible underlying mechanisms (Yaribeygi et al., 2019). Consequently, numerous genes such as INS, AKT1, IL-6, TP53, TNF, VEGFA, MAPK3, EGFR, EGF, and SRC have been revealed to be associated with the development of IR (Gao et al., 2020). The relatively prominent signaling pathways involved in the formation of IR are the pathways of insulin resistance, adipocytokine, insulin, PI3K-Akt, ERK, AMPK, and HIF-1 (Ozaki et al., 2016; Huang et al., 2018; Gao et al., 2020). In the glucose tolerance test, intragastric administration of a lychee seed water extractant remarkably decreased hyperinsulinemia and potentiated insulin sensitivity (Guo et al., 2003a). Another study indicated that lychee seed extracts could increase the quality of life of streptozotocin (STZ) combined with a high-fat diet–induced type 2 diabetes rats. Compared to the control group, the insulin resistance index in the lychee seed extract group was dramatically reduced, which in turn increased the insulin sensitivity index progressively (Man et al., 2016). The PI3K/AKT/mTOR signaling pathway makes essential contribution to the occurrence of IR. Activation of the PI3K/AKT/mTOR signaling pathway could improve insulin-induced glucose (Yin et al., 2017; Han et al., 2020). Lychee seed extracts significantly improved IR in a type 2 diabetes mouse model by elevating the expression levels of PI3K, AKT, and mTOR to trigger the PI3K/AKT/mTOR signaling pathway (Man et al., 2017). Recently, growing evidence has shown that microRNAs as crucial regulators of gene expression perform a critical role in the development of IR (Honardoost et al., 2014; Wen et al., 2014; Xihua et al., 2019). One study showed that the microRNA expression changed significantly in db/db mouse administered extract of lychee seed (0.015 g/d, i.g.) (Zhang et al., 2013). In addition, abundant studies have demonstrated that endoplasmic reticulum stress–induced pancreatic β-cell destruction is one of the vital mechanisms of progression for both insulin-dependent diabetes and non–insulin-dependent diabetes (Cnop et al., 2017). Endoplasmic reticulum stress can not only directly damage the insulin signaling pathway but also further promote IR in a variety of ways (Ozcan and Tabas, 2012; Dong et al., 2017). Experiments *in vitro* have confirmed that lychee semen effective constituents can significantly reduce the mRNA expression of glucose regulatory protein 78 (Grp78) (Liao et al., 2014; Li et al., 2015) which contributes to endoplasmic reticulum stress and the activation of unfolded protein response (UPR). Elevated pro-inflammatory cytokine tumor necrosis factor-α (TNF-α) and leptin levels have been demonstrated to be closely associated with IR (Ayina et al., 2016; Alzamil, 2020). In addition, plasma free fatty acid (FFA) is viewed as a potential factor to IR and disrupts insulin secretion (Bergman and Ader, 2000). Lychee seed extracts could improve insulin sensitivity by reducing the levels of TNF-α, hyper-leptinemia, and FFA in diabetic rats (Guo et al., 2004).

### Antioxidant Effect

Oxidative stress is induced by an imbalance between the production of free radicals and the antioxidant mechanisms, which is a well-known contributor to the pathogenesis and progression of diabetes *via* several molecular mechanisms, such as β-cell dysfunction and defects of the normal insulin signaling pathways (Yaribeygi et al., 2020). In addition, the excessive production of reactive oxygen species (ROS) inside the cell occupies a pivotal role in the onset of oxidative stress (Zhang et al., 2016). The body produces excess ROS, which is known to enhance nuclear factor (NF)-κB activity (Zheng et al., 2015), β-cell maturation, and apoptosis increase. In a 1,1-diphenyl-2-picrylhydrazyl (DPPH) radical scavenging assay, the total flavonoids of lychee seed showed a potent antioxidant activity (Ren et al., 2017). Moreover, lychee seed extracts could significantly accelerate the clearance of O_2_^−^ in the cerebrum of mice with diabetes induced by alloxan (Li et al., 2006). Malondialdehyde (MDA), an oxidative stress marker, is produced when ROS within cells oxidize unsaturated fatty acids (Kwiecien et al., 2014). Several animal studies revealed that lychee seed extracts remarkably improved the activity of superoxide dismutases (SODs) which was the central antioxidant defense system against O_2_^−^ (Fukai and Ushio-Fukai, 2011) and decreased MDA in animal models of diabetic rats (Pan et al., 1999; Guo et al., 2003b; Guo et al., 2004).

### Anti-Inflammatory Effect

The relationship between inflammation and diabetes has received extensive attention. It is believed that diabetes is a chronic inflammatory state (Wellen and Hotamisligil, 2005). Indeed, recent studies have emphasized and found substantial evidence that many inflammatory cytokines such as transforming growth factor beta 1 (TGF-β1) (Herder et al., 1984-2002; Olivieri et al., 2010; Shi et al., 2018), monocyte chemotactic peptide 1 (MCP-1) (Reddy et al., 2017), and macrophage migration-inhibitory factor (MIF) (Sánchez-Zamora and Rodriguez-Sosa, 2014; Abu El-Asrar et al., 2019) are reported to be responsible for the pathogenesis of the development of diabetes or diabetes complications. The currently available medical therapy mainly targets the underlying etiology. Hence, inhibition of excessive inflammatory responses might provide a potentially promising candidate for future therapeutics of diabetes. Lychee seed extracts could alleviate the inflammation reaction in rats with impaired glucose tolerance, which was associated with the downregulation expression of TGF-β1, MCP-1, and MIF (Qi, 2017). NF-κB is central to inflammatory responses and is tightly linked to various inflammatory diseases. Lychee seed extracts directly affected the mRNA levels of NF-κB, which prevented diabetes (Man et al., 2016). An experiment in diabetic nephropathy mice models has revealed that the saponin of lychee seed could delay the diabetic kidney inflammation development through inhibiting the expression of MCP-1 and intercellular cell adhesion molecule-1 (ICAM-1) protein in the kidney tissue, reducing the content of pro-inflammatory cytokines including interleukin-1β (IL-1β) and interleukin-6 (IL-6) in the serum (Qin, 2017).

### Lipid Regulation

Glucose and lipid metabolism are intrinsically related to one another in many aspects. Diabetic dyslipidemia is common in individuals with diabetes (Athyros et al., 2018). The pathophysiological mechanism of diabetic dyslipidemia is highly complex and multifactorial, yet accepted as a preponderant contributor in the occurrence of diabetic dyslipidemia is IR with an attendant increase in free fatty acid flux into the liver (Mooradian, 2009). The most predominant clinical presentation of the interaction is marked by elevated triglycerides (TGs), decreased high-density lipoprotein cholesterol (HDL-C), and predominance of small-dense low-density lipoprotein (LDL) (Parhofer, 2015). The saponin of lychee seed affected the lipid metabolism in dexamethasone (DX)-induced insulin-resistant rats by lowering the content of total cholesterol (TC), TG, and low-density lipoprotein cholesterol (LDL-C) (Guo et al., 2003b). Simultaneously, the potential lipid-modifying effect of lychee seed extracts was also demonstrated by the other two animal studies (Pan et al., 1999; Li et al., 2015).

### Kidney Protection Effect

Diabetic kidney disease (DKD), a severe microvascular complication of diabetes, is the primary cause of end-stage renal failure and the single strongest predictor of mortality in diabetic patients (Reidy et al., 2014; Thomas et al., 2016). Strict glycemic management dramatically reduces DKD morbidity, which suggests that metabolic disorders resulting from hyperglycemia, including changes in energy utilization and mitochondrial damage, exert a critical role in the disease progression (Reidy et al., 2014). Presently, multifactorial management of DKD primarily includes diet therapy, glucose-lowering therapy, lipid control, and preserving renal function (Selby and Taal, 2020). Despite various therapeutic strategies, the morbidity and mortality of DKD remain high throughout the world. Traditional Chinese herbal medicine can possess antidiabetic effects and improve renal function on DKD obviously. Research showed that saponin of lychee seed could reduce the blood glucose and ameliorate pathological damage and kidney lesions of diabetic nephropathy model rats through repressing the expression of inflammatory factors and attenuating inflammatory responses in kidney tissue (Qin, 2017). Out of many cytokines implicated in fibrosis, transforming growth factor-β1 (TGF-β1), fibronectin (FN), and collagen IV (Col IV) promoting extracellular matrix (ECM) accumulation are the most notorious (Downer et al., 1988; Chen et al., 2017). In the rat mesangial cells induced by high glucose and tumor necrosis factor-α (TNF-α), the total flavonoids of lychee seed distinctly decreased the protein expression of TGF-β1, FN, and Col IV, which indicated the total flavone might improve the diabetic nephropathy fibrosis process (Liu, 2016; Liu et al., 2016). In addition, the saponin of the lychee seed was confirmed to obviously reduce the content of IL-6 and IL-1β secreted by human glomerular mesangial cells (HMC) and decrease the secretion of ECM to slow down the sclerosis process of glomerulus (Zhang, 2016).

### Neuroprotection and Cognitive Function Improvement

Cognitive dysfunction is considered as a serious and common comorbidity or even a complication of diabetes (Biessels and Despa, 2018). They share common biological mechanisms including deficits in insulin signaling, neuroinflammatory pathways, mitochondrial (Mt) metabolism, the sirtuin-peroxisome proliferator-activated receptor-gamma coactivator 1α (SIRT-PGC-1α) axis, and Tau signaling (Zilliox et al., 2016). An animal test showed that the lychee seed extract fluid could protect the nervous system by significantly improving the transmit function of the cholinergic nervous system in the cerebrum of mice with diabetes induced by alloxan and accelerating the clearance of O_2_^−^ (Li et al., 2006). In another research, compared with the model group, amyloid beta (Aβ) and Tau deposition of the experimental rats in the medium- and high-dose lychee seed extract administration groups [1.39 and 2.78 g/(kg.d)] were significantly decreased (Zeng et al., 2016). Similarly, investigators have found that lychee seed extracts consisting of numerous ingredients such as adenosine, 5-hydroxymethyluridine, and 4-p-coumaroylquinic acid dramatically protected against neuronal damage and prevented the decline in the cognitive function through lowering serum glucose, ameliorating IR, and suppressing the aggregation of Aβ, Tau protein, and advanced glycation end products (AGEs) in the hippocampus of type 2 diabetes rats (Tang et al., 2018), while further study demonstrated that polyphenols derived from lychee seed inhibited hyperphosphorylated Tau through improving IR *via* upregulating IRS-1/PI3K/Akt and downregulating GSK-3β (Xiong et al., 2020).

**FIGURE 2:**
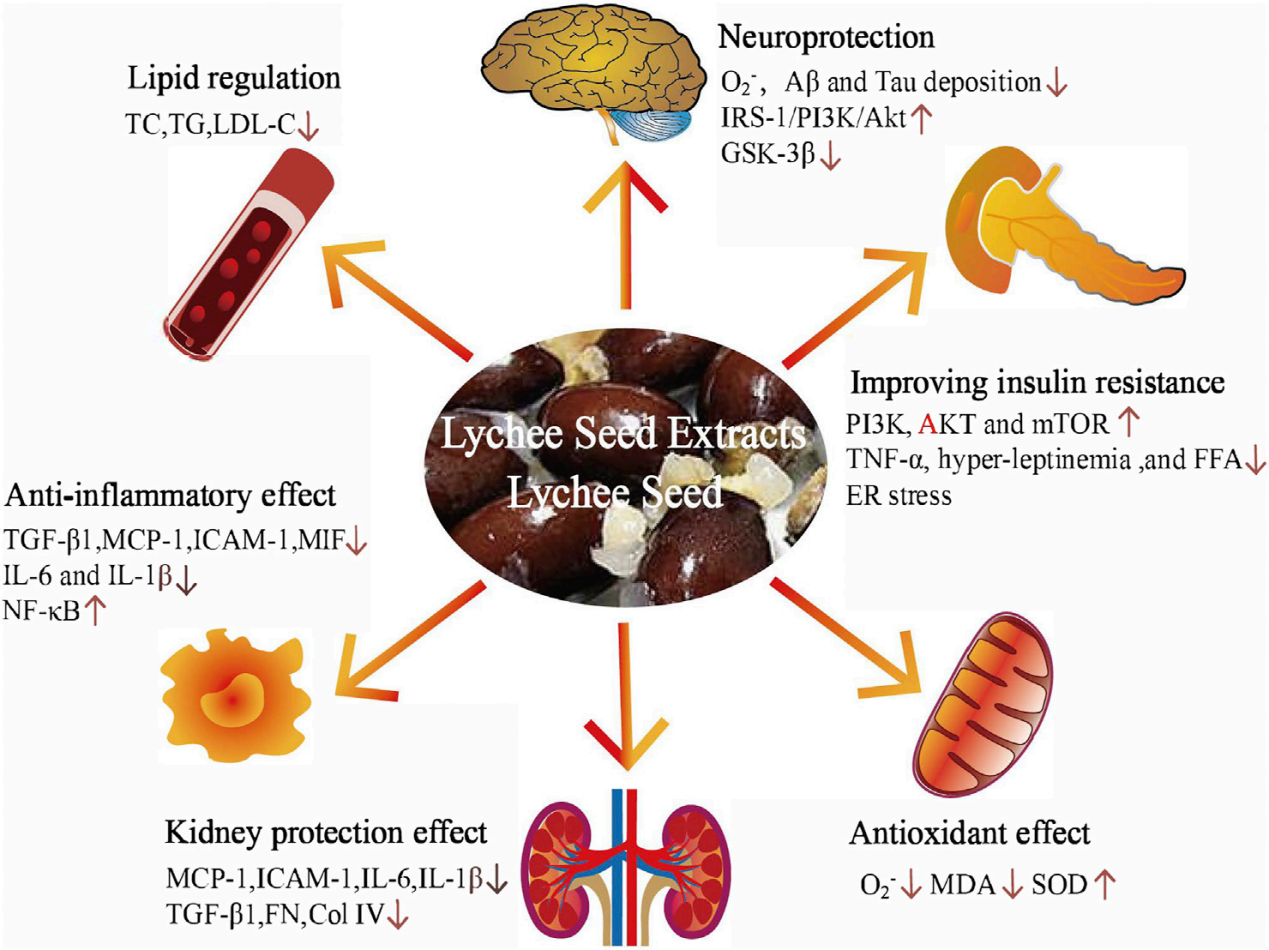
The underlying mechanism of hypoglycemic activity of lychee seed and lychee seed extracts. Amyloid beta (Aβ); tumor necrosis factor-α (TNF-α); free fatty acids (FFAs); endoplasmic reticulum (ER); malondialdehyde (MDA); superoxide dismutase (SOD); monocyte chemotactic protein 1 (MCP-1); intercellular cell adhesion molecule-1 (ICAM-1); interleukin-6 (IL-6); interleukin-1 (IL-1β); transforming growth factor-β1 (TGF-β1); fibronectin (FN); collagen IV (Col IV); macrophage migration-inhibitory factor (MIF); nuclear factor-κB (NF-κB); cholesterol (TC); triglyceride (TG); and low-density lipoprotein cholesterol (LDL-C).

Based on studies on diabetes and diabetic complication intervention with lychee seed *in vivo* and *in vitro*, the underlying hypoglycemic mechanisms of lychee seed are summarized in Figure 2.

## Conclusion and Perspective

The rising prevalence and financial burden of diabetes and its complications have made it one of the greatest health threats facing the 21st century. Although significant advances have been made toward a long-term therapeutic approach to treat diabetes, it is tough to control the blood glucose level precisely, and the use of oral hypoglycemic agents comes with many limitations, including side effects (gastrointestinal intolerance and myocardial events) (Nissen and Wolski, 2007; McCreight et al., 2016). Lychee seed as a natural source showed antidiabetic effects from lowering blood glucose to alleviating diabetic complications. Its beneficial effects have also been validated by several clinical observations (Zhang and Teng, 1985; Shen, 1991). Through the literature review, the underlying mechanisms, improving insulin resistance, antioxidant effect, anti-inflammatory effects, lipid regulation, kidney protection effect, and neuroprotection and cognitive function improvement of lychee seed in treating diabetes are also worth investigating. For further research of lychee seed within this field, several issues should be considered. An *in vitro* research showed that saponin of lychee seed had no effect on glycometabolism in an insulin resistance model of pepatocellular carcinoma (HepG2) cells (Qin, 2017), which may be related to the site of drug action. The impact of saponin of lychee seed on improving IR may not be effected in hepatocytes but in other peripheral tissues such as muscle and fat. Thus, the corresponding site of action of lychee seed needs to be explicitly investigated. Most of the elucidation of the antidiabetic mechanisms scratches only at the surface, and researchers need to probe deeper into analyzing the detailed molecular mechanisms of the effects of lychee seed intervention. Consequently, comprehensive and much more robust evidence is desperately needed. As outlined in the above review, although some clinical studies show positive results in the treatment of diabetes, large, double-blind, randomized, placebo-controlled, multicenter clinical trials are needed.

In conclusion, lychee seed might be developed as a multi-target agent and prescribed as a useful adjuvant to the current treatment for diabetes and especially diabetic complications. Despite the enormous therapeutic potential, further comprehensive investigation from bench to clinical reasearch is warranted.
